# Inter-Patient Congestive Heart Failure Detection Using ECG-Convolution-Vision Transformer Network

**DOI:** 10.3390/s22093283

**Published:** 2022-04-25

**Authors:** Taotao Liu, Yujuan Si, Weiyi Yang, Jiaqi Huang, Yongheng Yu, Gengbo Zhang, Rongrong Zhou

**Affiliations:** 1 School of Electronic and Information Engineering (SEIE), Zhuhai College of Science and Technology, Zhuhai 519041, China; ttliu19@mails.jlu.edu.cn (T.L.); yhyu19@mails.jlu.edu.cn (Y.Y.); zhanggb19@mails.jlu.edu.cn (G.Z.); zhourr19@mails.jlu.edu.cn (R.Z.); 2 College of Communication Engineering, Jilin University, Changchun 130012, China; yangwy19@mails.jlu.edu.cn; 3 Department of Biomedical Engineering, McGill University, Montreal, QC H3A 2B4, Canada; 4 College of Computer Science and Technology, Jilin University, Changchun 130012, China; huangjq19@mails.jlu.edu.cn

**Keywords:** congestive heart failure (CHF), electrocardiogram (ECG), Convolutional Neural Network (CNN), Vision Transformer, inter-patient scheme

## Abstract

An attack of congestive heart failure (CHF) can cause symptoms such as difficulty breathing, dizziness, or fatigue, which can be life-threatening in severe cases. An electrocardiogram (ECG) is a simple and economical method for diagnosing CHF. Due to the inherent complexity of ECGs and the subtle differences in the ECG waveform, misdiagnosis happens often. At present, the research on automatic CHF detection methods based on machine learning has become a research hotspot. However, the existing research focuses on an intra-patient experimental scheme and lacks the performance evaluation of working under noise, which cannot meet the application requirements. To solve the above issues, we propose a novel method to identify CHF using the ECG-Convolution-Vision Transformer Network (ECVT-Net). The algorithm combines the characteristics of a Convolutional Neural Network (CNN) and a Vision Transformer, which can automatically extract high-dimensional abstract features of ECGs with simple pre-processing. In this study, the model reached an accuracy of 98.88% for the inter-patient scheme. Furthermore, we added different degrees of noise to the original ECGs to verify the model’s noise robustness. The model’s performance in the above experiments proved that it could effectively identify CHF ECGs and can work under certain noise.

## 1. Introduction

There are more than 60 million people worldwide suffering from congestive heart failure (CHF) [1]. According to expert predictions, the prevalence of CHF will increase by 46% compared to 2012 by the year 2030 [2]. With an aging population trend, CHF has gradually become a serious health and safety issue [3] because this kind of disease is more harmful to older people [4]. CHF is caused by an insufficient blood supply from the heart to the body due to the ventricles’ low pumping or filling capacity. It is caused by myocardial damage due to various cardiovascular diseases [5]. During the onset, blood runs backwards or accumulates in the lungs, and that causes breathing difficulties and even dizziness. In severe cases, it can lead to potentially fatal acute heart failure. Therefore, early detection of CHF can prevent further damage to the heart, which is crucial and even lifesaving for patients with underlying heart failure.

Standard examination methods for CHF include echocardiography, cardiac magnetic resonance, and tubular angiography. However, these methods are complicated to perform and of a high cost. An electrocardiogram (ECG) contains complex cardiac information and is a simple, rapid, and non-invasive way to detect CHF [6]. Furthermore, because the ECG changes caused by CHF are subtle, the task of analyzing the ECG requires a considerable amount of time and intelligence from the professional physician. The computer-aided ECG classification system can realize automatic detection of CHF, saving doctors’ time and reducing the rate of misdiagnosis caused by fatigue, which is also of great significance for medical resource allocation [7].

Many works have proposed different ECG-based CHF detection algorithms, and the related works are summarized in Table 1. Orhan [8] adopted the equal frequency in amplitude and equal width in time (EFiA-EWiT) feature extraction method and used the linear regression (LR) classifier to achieve a CHF recognition accuracy of 99.33%. Kamath [9] used detrended fluctuation analysis (DFA) to distinguish the ECG between normal and CHF patients and obtained an accuracy of 98.2%. Sudarshan et al. [10] proposed a dual-tree complex wavelet transform (DTCWT) method for extracting statistical features in ECG signals and used feature ranking technology to send the highest-ranked salient features into k-nearest neighbors (KNN) as well as a decision tree (DT) for classification, achieving a recognition accuracy of 99.86%. Acharya et al. [11] developed an 11-layer CNN for the automatic feature extraction and classification of ECGs, which gained 98.97% accuracy. Darmawahyuni et al. [12] built an LSTM-based RNN structure model for identifying CHF, which reached 99.86% accuracy. Naik et al. [13] achieved 100% accuracy for normal and CHF heartbeats using the VGG16 network.

Although many studies have realized outstanding achievements in CHF detection and even obtained an accuracy as high as 100%, there is still room for improvement. First, the experiments of these works were all conducted under an intra-patient scheme, which can lead to the indeterminate performance of the model when receiving data from new individuals. Under the inter-patient scheme, the model detects ECGs from unknown ones, significantly increasing the demands on the model’s ability for feature extraction and generalization. Therefore, the inter-patient test has more practical significance. Additionally, because the ECG signals are vulnerable to noise interference during acquisition, the quality of ECGs differs under different acquisition conditions, increasing the difficulty of detection. However, the above work did not consider the noise robustness of the model. Therefore, accurate CHF detection for ECG signals with different noise levels can be adapted to different scenarios.

To address the above problems, the ECG-Convolution-Vision Transformer Network (ECVT-Net) is proposed to achieve a high-performance automatic CHF detection. The convolutional neural network (CNN), as a classic network model for image processing, has been proved in related research for effectively understanding the characteristic information of ECG signals [11,14,15]. However, its most disappointing disadvantage is that it cannot capture long-term dependencies due to the limited size of the receptive field [16]. A Transformer [17] is proposed in natural language processing (NLP), which extracts the correlation between words in the text sequence through the multi-head attention mechanism to achieve an excellent data fitting ability. The Vision Transformer (ViT) [18] splits and flattens the image and sends it to the Transformer, completing the image-to-sequence conversion. It realizes the extraction of the correlation between the features at different locations on the images and has shown an excellent feature mining ability [19,20,21]. As a 1D time series, an ECG has sequence properties similar to text sequences, so that its correlation at different positions in the time dimension can be extracted by ViT.

The ECVT-Net combines the advantages of CNN and ViT to achieve a high-performance ECG-based CHF classification system. First, CNN is used to perform the preliminary feature extraction on ECGs, capturing detailed local features of the signal. These features are then segmented and fed into the ViT, which could mine the correlation information between feature segments, reflecting the relationship between the heart states at different time periods. The feature map is finally sent to the classifier of the combination of multilayer perceptron (MLP) and SoftMax to determine whether the ECG is from a CHF patient or not. In addition, both models are less sensitive to noise due to the CNN’s ability to remove local noise and the ViT’s emphasis on global correlation [22,23,24].

The rest of this paper is organized as follows: Section 2 presents the database details used in the study and the experimental data grouping method. The implementation details of ECVT-Net are provided in Section 3. Section 4 introduces the experimental results and discusses the comparison with the methods in existing publications. Finally, Section 5 is the conclusion drawn by this research.

## 2. Materials

### 2.1. Databases

**Figure 1:**
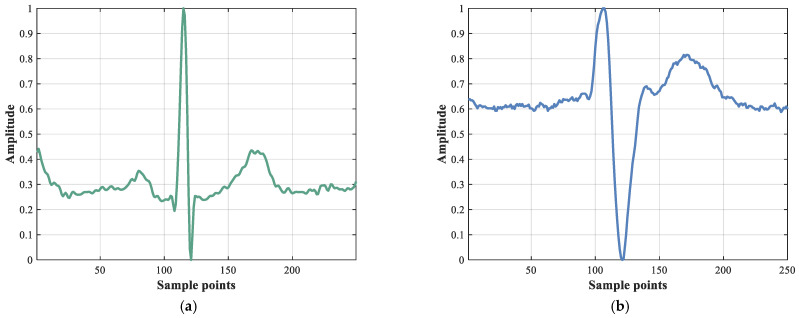
ECG waveforms from the BIDMC database and MITNSR database. (**a**) Normal heartbeat; (**b**) CHF heartbeat.

In this study, two commonly used ECG databases downloaded from PhysioNet [25] are used to verify the performance of the ECVT-Net, and both have R point information and disease labels marked by experts. The normal ECGs were obtained from the MIT-BIH normal sinus rhythm database (MITNSR) [25], and the CHF ECGs were adopted from the BIDMC congestive heart failure database (BIDMC) [26]. Figure 1 illustrates the two types of heartbeat waveforms.

1. The MITNSR database includes ECG recordings from 18 people in normal sinus rhythm, with a sampling frequency of 128 Hz.
2. The BIDMC database contains ECG recordings from 15 patients with severe CHF, sampled at 250 Hz.

The first 2000 heartbeats in each patient data from the above database were adopted as experimental data. The details for each database are summarized in Table 2.

### 2.2. Data Grouping

**Figure 2:**
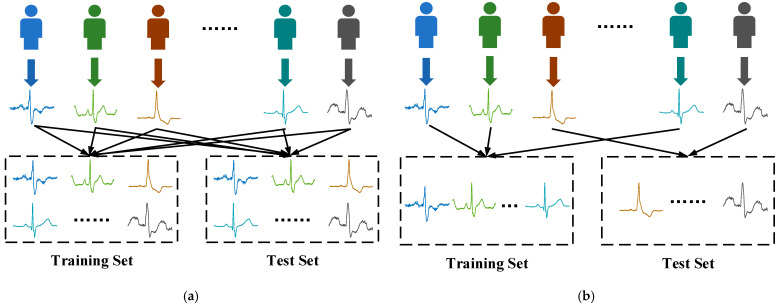
Principles of inter-patient and intra-patient schemes. (**a**) Intra-patient scheme; (**b**) Inter-patient scheme.

To thoroughly verify the model’s generalization performance, we designed both intra-patient and inter-patient experimental protocols. The mechanisms of these two schemes are shown in Figure 2.

1. The Intra-patient scheme: randomly divided the heartbeats of the same patient into training and test sets. To reduce the chance of the experiment, this scheme adopts a ten-fold cross-validation.
2. The Inter-patient scheme: different patients in training set and test set.

The detailed configurations of the above two experimental schemes (patient ID, number of beats distribution) are shown in Table 3.

## 3. Methods

**Figure 3:**
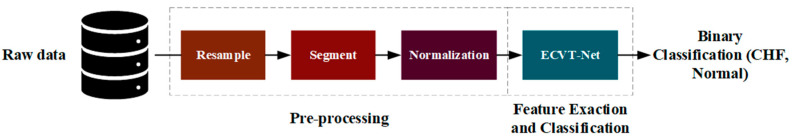
Overall flow of the proposed method.

In this part, the proposed ECG detection system will be introduced in detail according to the algorithm steps, and the overall flow chart is shown in Figure 3.

### 3.1. Pre-Processing

**Figure 4:**
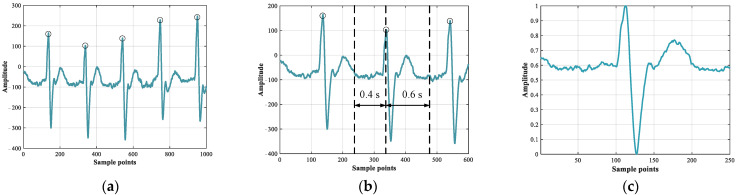
Waveform of ECG during pre-processing. (**a**) Raw data (R points are given in the database); (**b**) Heartbeat segment; (**c**) Heartbeat after normalization.

Due to that the disease labels are annotated at each heartbeat cycle, the long original ECG data needs to be pre-processed into multiple segments. As shown in Figure 3, the pre-processing process consists of three steps: resampling, segmentation, and normalization. Figure 4 shows the changes of the ECG waveform during the pre-processing process. First, to ensure that the data from different databases are sampled with equal frequency, the data from MITNSR need to be resampled to 250 Hz. Secondly, we take the R point as the center and take 0.4 s before the R point to 0.6 s after the R point as a heartbeat interval. Finally, a max–min normalization method is applied to normalize the amplitude interval of each heartbeat to the [0, 1] interval. The purpose of normalization is to solve the amplitude scaling problem and eliminate the singular value effect so that the model training can be better.

### 3.2. Feature Extraction and Classification (ECVT-Net)

#### 3.2.1. Overview

**Figure 5:**
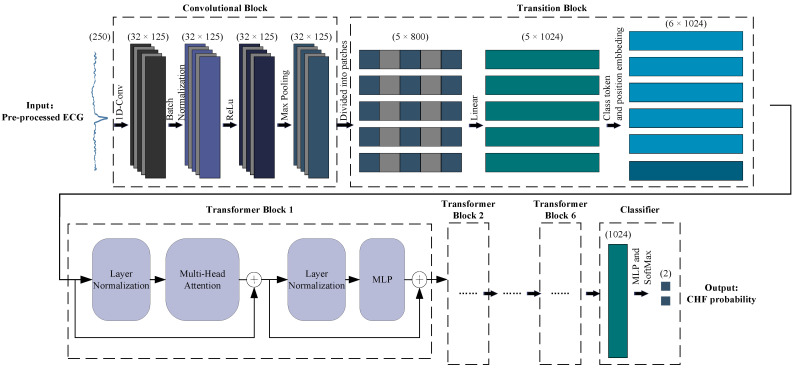
Pipeline of ECVT-Net for ECG feature extraction and classification.

After pre-processing, the heartbeats will be input into ECVT-Net. The flow chart of ECVT-Net is shown in Figure 5. ECVT-Net is inspired by both CNN and Vision Transformer, which combines their excellent feature extraction capabilities. With a superb representation learning ability, CNN sequentially convolves with signals at different positions through convolution kernels. Still, it lacks the comprehension of the dependencies between distant features in long-term sequences. Therefore, ViT is introduced to enhance the sequence modeling capability. ECVT-Net consists of four parts: the convolutional block, the transition block, the Transformer block, and the classifier. The details of deployment will be introduced one by one below.

#### 3.2.2. Convolutional Block

As the first part of the ECVT-Net, the convolutional block adopts 1D convolution to achieve preliminary learning of pre-processed ECGs. This stage aims to improve the local correlation of features in the time dimension and remove some redundant features. At the same time, the application of convolutional layers also improves the noise robustness, making the model more suitable for the application scenarios of ECG classification tasks. After the convolutional layer, the batch normalization (BN) [27], the rectified linear unit (ReLU) [28], and the max-pooling layer are added to optimize the feature distribution and improve the model’s performance. Among them, BN normalizes the output of each batch to the [0, 1] interval. ReLu maps the output to the linear interval. Max pooling takes the maximum value in the adjacent interval. These three operations are applied to make the features more compact, improve the efficiency of gradient propagation, and prevent overfitting.

#### 3.2.3. Transition Block

To connect CNN and ViT effectively, at this stage, feature vectors from convolutional block are split into fixed-size sequences and go through a layer of trainable linear transformation to generate five trained patch embeddings. The “class token” and the position embeddings are then added to these patch embeddings so that the model can learn and recognize location information more efficiently. In addition, the dimensionality reduction operation is omitted compared to the original ViT [18] since an ECG is a 1D time series, which reflects the adaptability of Transformer to time series.

#### 3.2.4. Transformer Block

Next, the feature patches will be input into six consecutive Transformer blocks. As shown in Figure 5, the Transformer block includes a multi-head attention layer and an MLP layer. Layer Normalization (LN) [29] is applied ahead of each layer, and residual connections are used following each layer. The mathematical representation of the Transformer block is as follows:

(1) $$z_{L}^{0}=[x_{cls};x_{p}^{1}E;x_{p}^{2}E;\cdots ;x_{p}^{n}E]+E_{pe}$$

(2) $$z_{L}^{\prime }=\mathrm{MHA}(\mathrm{LN}(z_{L-1}))+z_{L-1}$$

(3) $$z_{L}=\mathrm{MLP}(\mathrm{LN}(z_{L}^{\prime }))+z_{L}^{\prime }$$

where $z_{L}^{0}$, $z_{L}^{\prime }$, and $z_{L}$ represent the input, intermediate layer feature, and output of the *L*-th Transformer block, respectively. $x_{cls}$ is the class token. Each of $x_{p}^{1},x_{p}^{2},\ldots ,x_{p}^{n}$ represents *n* feature patches. *E* is the linear transformation. $E_{pe}$ is the position embedding. MHA is short for multi-head attention, and MLP is a feedforward neural network with two hidden layers.

Multi-head attention is multiple independent self-attention operations. In the self-attention process, each feature patch is mapped to three matrices: *Q* (Query), *K* (Key), and *V* (Value) through linear transformation, and the output is the result of multiplying V by the product weighted by *QK*. This operation expresses the association information between each feature block and reflects the dependencies between the various periods of the ECG. The multi-head attention is similar to multiple convolution kernels in CNN. It runs numerous attention operations to learn features simultaneously, so that the algorithm can mine ECG information in more feature space and capture more comprehensive feature details. The formula is as follows:

(4) $$\mathrm{MHA}(Q,K,V)=\mathrm{Concat}(head_{1},\ldots ,head_{h})$$

(5) $$head_{i}=\mathrm{Attention}(Q_{i},K_{i},V_{i})$$

(6) $$\mathrm{Attention}(Q,K,V)=\mathrm{softmax}(\frac{QK^{T}}{\sqrt{d}})V$$

where Concat means concatenation. $head_{i}$ represents the *i*-th self-attention operation in MHA, and *d* is the dimension of the *Q* and *K* matrices.

#### 3.2.5. Classifier

Classification is conducted by a combination of a trainable MLP, and Softmax [30]. Among them, MLP consists of LN and a fully connected layer. Softmax normalizes the output from MLP to (0, 1) interval, which gives the probability of disease and improves the efficiency of updating weights during model training.

### 3.3. Evaluation Indicators

Three basic metrics are adopted in this study to reflect the model’s performance and comparison with related work: accuracy (Acc), precision (Pr), and sensitivity (Se), which can be obtained by the confusion matrix according to Equations (7)–(9). Among them, true positive (TP) indicates that the model gives the correct type of heartbeat, while true negative (TN) implies that the heartbeats of the negative type are correctly identified. False negative (FN) indicates that the model wrongly identifies the positive heartbeats as a negative class. False positive (FP) refers to the incorrect identification of a negative heartbeat as a positive one.

(7) $$\mathrm{Acc}=\frac{\mathrm{TP}+\mathrm{TN}}{\mathrm{TP}+\mathrm{FP}+\mathrm{TN}+\mathrm{FN}}\times 100\%$$

(8) $$\mathrm{Pr}=\frac{\mathrm{TP}}{\mathrm{TP}+\mathrm{FP}}\times 100\%$$

(9) $$\mathrm{Se}=\frac{\mathrm{TP}}{\mathrm{TP}+\mathrm{FN}}\times 100\%$$

## 4. Results and Discussion

### 4.1. Experimental Setup

The experiments were implemented in MATLAB R2021b and a PyCharm 2021 environment using a personal laptop with an AMD Ryzen 9 5900HX CPU (@3.30GHz), an NVIDIA GeForce RTX 3060 Laptop GPU, and 16 GB of RAM. The proposed ECVT-Net was developed with the Python (3.8) programming language and the PyTorch deep learning framework.

**Figure 6:**
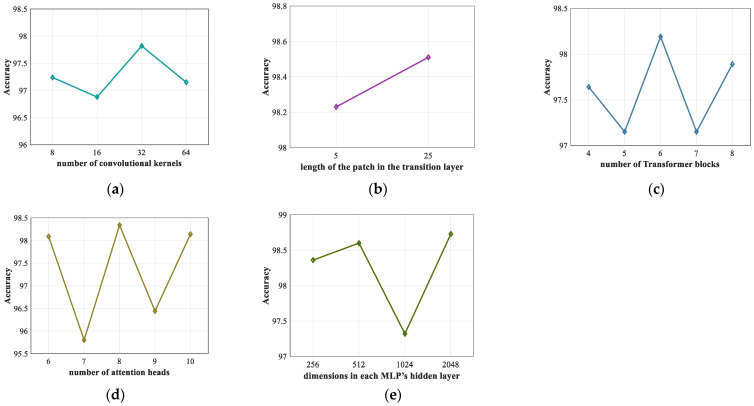
Impact of the structural parameters of ECVT-Net. (**a**) Number of convolutional kernels; (**b**) Length of the patch in the transition layer; (**c**) Number of Transformer blocks; (**d**) Number of attention heads; (**e**) Dimensions in each MLP’s hidden layer.

During the model training, Stochastic Gradient Descent (SGD) and Cross entropy loss function are adopted to optimize the weights. For the modifiable hyperparameters in the model, under the intra-patient scheme, we conducted multiple experiments to find the best parameter settings through the grid search method. First, we tested the impact of the network structure parameters on the model performance, and the results are presented in Figure 6. Then, we tested the effects of other key parameters in model learning, such as batch size, epoch, and learning rate. The detailed parameter settings are shown in Table 4.

### 4.2. Results on the Intra-Patient Scheme

The intra-patient scheme was performed to initially observe the adaptability of the model to ECG data. Table 5 shows the performance of the ECVT-Net for the intra-patient experimental protocol with a 99.96% accuracy rate, and only 27 beats were misclassified out of 66,000 beats. The excellent results are due to that the data in the intra-patient test set contains data from the same patients as the training set, making the two sets highly similar in distribution, causing the model to overfit the features of each patient. In addition, other indicators are above 99.95%, which preliminarily shows the model’s adaptability to ECG, but also reflects the necessity of an inter-patient scheme.

### 4.3. Results on the Inter-Patient Scheme

Table 6 shows the ECVT-Net’s performance under the inter-patient pattern. Such grouping can fully verify the generalization performance of the model and is closer to the clinical application scenario. In this experiment, the model reached an accuracy of 98.88%, except for the Pr of the CHF class that obtained 97.86%; all other indicators were above 98%. Additionally, the model achieves high indicators of 99.82 and 99.79% for the Pr of normal beats and Se of CHF beats, respectively.

### 4.4. Results under Different Noise Levels

**Figure 7:**
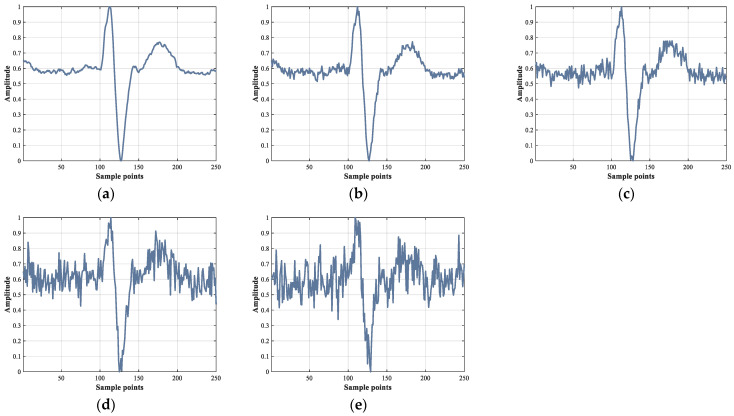
The heartbeat waveforms at different SNRs. (**a**) SNR = ∞ dB (Raw data after pre-processing); (**b**) SNR = 24 dB; (**c**) SNR = 18 dB; (**d**) SNR = 12 dB; (**e**) SNR = 6 dB.

To verify the performance of the ECVT-Net under conditions of different hospitals and different clinical scenarios, we tested the performance of the ECVT-Net for different ECG signal qualities. The awgn function in MATLAB was used to add Gaussian white noise with different signal–noise ratios (SNRs) to the raw data in the inter-patient protocol test set. The heartbeat waveforms at different SNRs are shown in Figure 7.

Table 7 shows the classification performance of the ECVT-Net for ECG signals with different SNRs. As the SNR of ECGs decreases, the model’s performance deteriorates. It is worth noting that the model’s performance degrades slowly when the SNR is not higher than 12 dB, and the indicators are around 95% when the SNR is 12 dB.

### 4.5. Ablation Experiment

Besides a comprehensive performance evaluation of the model, we conduct ablation experiments on different components of the convolutional block under the inter-patient scheme and compare it with the CNN model and the original 1D ViT model. The classification performance is shown in Table 8. Among the results, we use the classic 1D Alex-Net [31] as a comparison model to simulate the model’s adaptability to ECGs in the case of only CNN. Results show that CNN has better adaptability to ECG than ViT, and simply combining the two will not help the model’s performance much. After adding BN, ReLu, and pooling layers, all the indicators of the model have reached more than 98.8%, which is a significant improvement.

### 4.6. Comparison and Performance Analysis

The motivation of this paper is to develop an ECG-based CHF diagnostic model that is more adaptable to application scenarios and robust to noise. In this paper, the ECVT-Net is proposed based on a CNN and Transformer to identify ECG signals from normal people and CHF patients. In this section, we analyze the model’s performance and compare it with the methods from existing publications. Table 9 summarizes the research work using the same database as this paper.

To verify the model’s generalization, we set up both intra-patient and inter-patient experimental protocols, while other literature only performed intra-patient experiments [8,9,12]. The experimental results show that the ECVT-Net has excellent adaptability to ECG characteristics. First, the performance in intra-patient experiments is higher than the experimental results in the current literature, and the generalization can satisfy inter-patient tasks. Secondly, the existing literature lacks the consideration of anti-noise performance, and we tested the model’s performance for ECGs with different qualities. The results demonstrated that the model is robust to noise. In addition, the effectiveness of a CNN and ViT combination and the contribution of different components are also verified by ablation experiments.

The excellent performance of the ECVT-Net is due to that the model’s integration of the excellent representation capabilities of a CNN and a ViT, which fully exploits the high-dimensional abstract features of ECGs and forms a rich feature space. First, since ECG is a 1D time series, we adopted a combination of 1D convolution and ViT in modeling, which avoids folding or changing the dimension of the input signal and maintains the essential characteristics of the time series. A CNN pays attention to the details of local features of ECG signals and reduces local redundancy. A ViT focuses on the global dependencies of ECG features and has a strong modeling ability for sequence information. The hybrid architecture of the two algorithms improves the local and global compactness of the features and builds an efficient semantic representation model for ECGs. The generalization ability of the ECVT-Net benefits from its powerful feature mining ability, which makes it perform well under the inter-patient scheme. Furthermore, this study tests different variants of the combination of CNN and ViT, focusing on feature flow in transition layers between the two, validating the effectiveness of the modeling. More importantly, the original intention of choosing these two algorithms is that they have excellent noise robustness and can adapt to different clinical scenarios.

In summary, the ECVT-Net proposed in this paper mainly has the following characteristics:

1. High accuracy;
2. Automatic CHF detection model combining a CNN and a ViT;
3. Good generalization performance that can work under the scheme between patients;
4. Robust to noise;
5. No manual feature extraction.

## 5. Conclusions

The early detection of CHF is essential for potential patients to prevent exacerbations and reduce risk. This study proposed the ECVT-Net combining a CNN and a ViT to realize the automatic identification of CHF based on ECGs. Under the inter-patient pattern, the data distributions of training and test sets are close to the application scenario; the Acc, Pr, and Se of the proposed system reached 98.88, 98.84, and 98.94%, respectively. In addition to that, the model also achieves stable results in experiments with different levels of noise interference. Therefore, our model has the potential to work in a variety of clinical scenarios. In future work, we will expand the data scale, test more disease types, and aim to generalize the model to more clinical scenarios.

## Figures and Tables

**Table 1:** Relevant research work on CHF detection.

Author (Year)	Method	Database	Results
Orhan (2013) [8]	EFiA-EWiT, and LR	CHF: BIDMC Normal: MITNSR	Acc: 99.33% Se: 99.36%
Kamath (2015) [9]	DFA	CHF: BIDMC Normal: MITNSR	Acc: 98.20% Se: 98.40%
Sudarshan et al. (2017) [10]	DTCWT, DT, and KNN	CHF: BIDMC Normal: Fantasia, MITNSR	Acc: 99.86% Se: 99.78%
Acharya et al. (2019) [11]	11-layer deep CNN	CHF: BIDMC Normal: Fantasia	Acc: 98.97% Se: 98.87%
Darmawahyuni et al. (2020) [12]	LSTM	CHF: BIDMC Normal: MITNSR	Acc: 99.86% Pr: 99.86% Se: 99.85%
Naik et al. (2021) [13]	VGG16	CHF: BIDMC Normal: Fantasia	Acc: 100%

**Table 2:** Details of databases.

Database	ECG Type	Sampling Rates (Hz)	ID	Individual (Sex, Age)	Total Heart Beats Used
MITNSR	Normal	128	16265, 16272, 16273, 16420, 16483, 16539, 16773, 16786, 16795, 17052, 17453, 18177, 18184, 19088, 19090, 19093, 19140, 19830	5 men (aged 26~45), 13 women (aged 20~50)	36,000
BIDMC	CHF	250	chf01~chf15	11 men (aged 22~71), 4 women (aged 54~63)	30,000

**Table 3:** Customized experimental implementation.

Scheme	Category	Training Set		Test Set	
		Patient ID	Number of Heartbeats	Patient ID	Number of Heartbeats
Intra-patient	Normal	Mixed	32,400	Mixed	3600
(Cross-validation)	CHF	Mixed	27,000	Mixed	3000
Inter-patient	Normal	16265, 16272, 16273, 16420, 16483, 16539, 16773, 16786, 16795, 17052,	20,000	17453, 18177, 18184, 19088, 19090, 19093, 19140, 19830	16,000
	CHF	chf01~chf08	16,000	chf09~chf15	14,000

**Table 4:** Detailed settings of ECVT-Net.

Parameter	Value	Alternative List	Meaning
Batch size	512	(32, 64, …, 1024)	Quantity of heartbeats per batch
Epoch	100	(50, 100, 150)	Number of training iterations
Feature channel	32	(8, 16, 32, 64)	Number of convolutional kernels
Patch length	25	(5, 25)	Length of the patch in the transition layer
Depth	6	(4, 5, 6, 7, 8)	Number of Transformer blocks
Head	8	(6, 7, 8, 9, 10)	Number of attention heads
MLP dim	2048	(256, 512, 1024, 2048)	Dimensions in each MLP’s hidden layer
Learning rate	0.001	(0.1, 0.01, 0.001)	Learning rate of the optimizer

**Table 5:** Confusion matrix for the intra-patient experiment after ten-fold cross-validation.

Original/Predicted	Normal	CHF	Pr (%)	Se (%)
**Normal**	35,987	13	99.96	99.96
**CHF**	14	29,986	99.96	99.95
Average (%)			99.96	99.96
Acc (%)			99.96	

**Table 6:** Confusion matrix for the inter-patient experiment.

Original/Predicted	Normal	CHF	Pr (%)	Se (%)
**Normal**	15,694	306	99.82	98.09
**CHF**	29	13,971	97.86	99.79
Average (%)			98.84	98.94
Acc (%)			98.88	

**Table 7:** Model’s performance at different SNRs under inter-patient scheme.

SNR (dB)	ACC (%)	Pr (%)	Se (%)
∞	98.88	98.84	98.94
24	98.60	98.56	98.64
18	97.99	97.98	97.99
12	94.69	95.04	94.45
6	87.97	89.99	87.24

**Table 8:** Results of Ablation experiment under inter-patient scheme.

Model	Acc (%)	Average	
		Pr (%)	Se (%)
CNN (1D Alex-Net)	97.99	97.98	97.98
ViT	95.36	95.55	95.19
Conv + ViT	95.45	95.62	95.29
Conv + BN + ViT	98.32	98.29	98.35
Conv + BN + ReLu + ViT	98.19	98.17	98.20
Conv + BN + ReLu + Pooling + ViT (ECVT-Net)	98.88	98.84	98.94

**Table 9:** Summary of the studies on CHF recognition using ECG data obtained from BIDMC and MITNSR database.

Author	Method	Results	
		Intra-Patient	Inter-Patient
Orhan. [8]	EFiA-EWiT, and LR	Acc: 99.33% Se: 99.36%	
Kamath. [9]	DFA	Acc: 98.20% Se: 98.40%	
Darmawahyuni et al. [12]	LSTM	Acc: 99.86% Pr: 99.86% Se: 99.85%	
Ours	ECVT-Net	Acc: 99.96% Pr: 99.96% Se: 99.96%	Acc: 98.88% Pr: 98.84% Se: 98.94%

## Data Availability

The dataset used in this study is publicly available in the PhysioNet at https://physionet.org/ (accessed on 27 January 2022).
